# Bandwidth-Enhancement Single-Patch Antenna with Dual-Beam Pattern via Seven-Mode Operation

**DOI:** 10.3390/s26134067

**Published:** 2026-06-26

**Authors:** Shixi Huang, Nengwu Liu, Qilong Yan

**Affiliations:** 1 School of Communication Engineering, Xidian University, Xi’an 710071, China; 23009100922@stu.xidian.edu.cn; 2 National Key Laboratory of Radar Detection and Sensing, Xidian University, Xi’an 710071, China; nwliu@xidian.edu.cn

**Keywords:** microstrip patch antenna, wide bandwidth, multi-mode resonances, slot loading, bandwidth enhancement

## Abstract

**Highlights:**

**What are the main findings?**

- A compact in-phase-fed microstrip patch radiator employing a single element is developed to realize dual-beam radiation through the coordinated excitation of seven resonant modes.
- An operating impedance band from 4.4 to 6.9 GHz is obtained, while the antenna profile remains as low as approximately 0.056λ_0_.

**What are the implications of the main findings?**

- These findings demonstrate that appropriate reallocation of multiple higher-order resonances can effectively broaden the operating bandwidth of compact patch radiators.
- The proposed design provides a useful approach for developing wideband low-profile antennas with stable radiation performance for wireless and radar applications.

**Abstract:**

This paper presents a low-profile wideband in-phase-fed microstrip patch antenna (MPA) operating with TM_60_, TM_04_, TM_62_, TM_24_, TM_44_, TM_82_, and TM_80_ modes. First, a rectangular in-phase-fed MPA is theoretically analyzed to clarify how these seven modes can be effectively utilized for bandwidth enhancement. Then, a transverse slot is incorporated into the patch to progressively move the resonance of the TM_04_, TM_24_, and TM_44_ modes toward that of the TM_60_ mode. Afterward, a pair of longitudinal slots is further employed to bring the TM_62_, TM_82_, and TM_80_ modes closer to the TM_60_ mode. In addition, another pair of longitudinal slots is introduced to improve the impedance matching performance. In this way, the seven modes are properly redistributed and grouped within the desired frequency range, thereby enabling broadband operation. Finally, an antenna prototype is manufactured and tested. The experimental results demonstrate that the antenna provides an impedance bandwidth covering 4.4–6.9 GHz while maintaining stable radiation performance, which is about 25 times wider than that of the conventional counterpart. More importantly, the antenna profile is as low as approximately 0.056λ_0_.

## 1. Introduction

Radar sensing and wireless communication systems require flexible directional radiation apertures for target detection, imaging, and high-capacity links [1,2]. Dual-beam antennas can provide two spatial beams from one aperture, making them suitable for multi-zone coverage and multi-target operation [3,4]. In the past few decades, researchers have proposed many design methods for dual-beam broadband low-profile microstrip antennas. However, simultaneously achieving low profile, wide bandwidth, and stable dual-beam radiation remains challenging for microstrip patch antennas.

In recent years, several effective methods have been reported to address this issue. The first approach is slot loading. In [5], a U-slot microstrip antenna operating at a higher-order mode achieved dual-beam radiation with a relatively low profile of 0.058λ_0_, but its bandwidth was only 11.8%. In [6], a single-feed dual-band U-slot antenna obtained bandwidths of 7.3% and 12.7% at the lower and higher bands, respectively, but its overall height reached about 0.16λ_0_. In [7], a dual-beam filtering patch antenna using a slotted patch, metal strip, and pins achieved a bandwidth of 23.5% with a profile of 0.058λ_0_, but the bandwidth was still insufficient for wider-band applications. Therefore, although slot loading can improve impedance matching and introduce additional resonances, it is still difficult to achieve both a very wide bandwidth and low profile by this method alone.

The second approach is metasurface loading. In [8], a low-sidelobe dual-beam antenna based on a metasurface achieved a relative bandwidth of about 30.3%. However, the feed was located 90 mm above the metasurface, corresponding to about 4.95λ_0_ at the center frequency, resulting in a high profile. In [9], a single-feed dual-polarized dual-beam holographic metasurface antenna achieved a low profile of about 0.066λ_0_, but its impedance bandwidth was only about 8%. Therefore, although metasurface loading is effective for dual-beam radiation, it remains difficult to simultaneously realize a low profile and sufficiently wide bandwidth.

In addition to the methods discussed above, multi-mode operation is another promising approach. In [10], a cross-shaped probe was used to excite the TM_02_ mode with a profile of about 0.10λ_0_, but the bandwidth was only 13.2%. In [11], a dual-band slot patch antenna achieved relatively low profiles of about 0.089λ_0_ at 3.8 GHz and 0.117λ_0_ at 5.0 GHz, but its measured bandwidths were only 8.0% and 9.9%. These results indicate that higher-order modes are useful for realizing low-profile dual-beam antennas. However, most existing designs employ only two main radiation modes, and thus, the achievable bandwidth is still restricted.

This paper presents a low-profile broadband in-phase-fed MPA based on the cooperative operation of seven higher-order resonant modes for bandwidth enhancement and dual-beam radiation. A rectangular in-phase-fed MPA with multi-mode operation is first investigated, and the selected modes are then redistributed to form a wide operating band. Specifically, two transverse slots are used to lower the resonant frequencies of TM_04_, TM_24_, and TM_44_, while two longitudinal slots are introduced to shift TM_60_, TM_62_, TM_82_, and TM_80_ toward the lower-frequency region. Finally, an outer longitudinal slot pair is added along the lateral boundaries of the radiator to optimize impedance matching. Consequently, a fractional bandwidth of 43.4% is achieved with a low profile of 0.056λ_0_, while stable dual-beam radiation patterns are effectively preserved.

## 2. Materials and Methods

**Figure 1:**
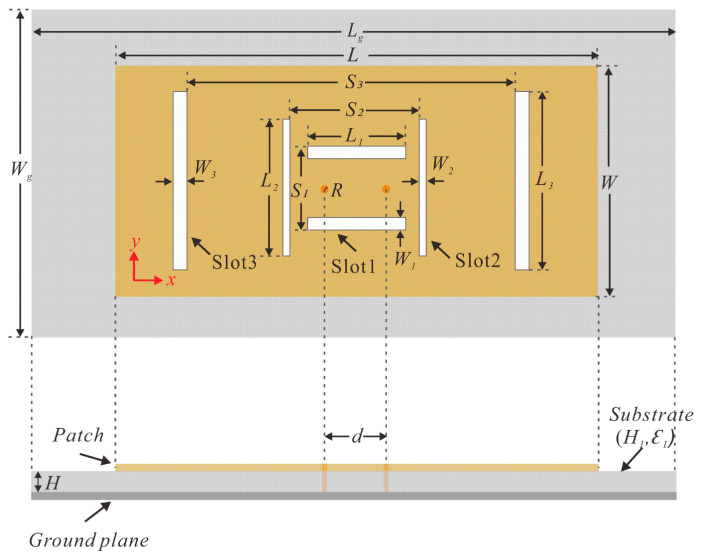
Geometry of the proposed in-phase-fed antenna.

This section introduces the working principle of the proposed in-phase-fed microstrip patch antenna and explains how the seven resonant modes, namely TM_60_, TM_04_, TM_62_, TM_24_, TM_44_, TM_82_, and TM_80_, are utilized to enhance the impedance bandwidth. The structural layout of the antenna is depicted in Figure 1. The configuration includes a rectangular radiating patch of size *L* × *W*, a dielectric substrate with dimensions *L*_g_ × *W*_g_, and a metallic ground plane identical in size to the substrate. The substrate thickness is *H* = 3 mm, with a loss tangent of about 0.01 and a relative permittivity of *ε*_r_ = 2.2. The feed radius is denoted as *R*. The ground plane is positioned underneath the substrate. A transverse slot (Slot1) is embedded in the patch to tune the resonant frequencies of the TM_04_, TM_24_, and TM_44_ modes, bringing them progressively closer to the TM_60_ mode. In addition, a pair of longitudinal slots (Slot2) is incorporated into the patch to adjust the resonant frequencies of the TM_62_, TM_82_, and TM_80_ modes, such that these modes also approach TM_60_. Furthermore, another pair of longitudinal slots (Slot3) is introduced to improve the input impedance behavior, enabling the impedance loop to move toward the central region of the Smith chart for enhanced probe-fed matching. Through this design, the seven resonant modes are arranged in close proximity over a wide frequency range and jointly contribute to radiation, thereby achieving an enhanced impedance bandwidth. The vector network analyzer AV3672 is adopted to conduct the experimental measurement of the fabricated antenna prototype. The proposed antenna is investigated and optimized with HFSS 2023, and the obtained simulation results are discussed in the following sections. The main geometrical parameters of the antenna configuration in Figure 1 are listed in Table 1.

### 2.1. Multi-Mode Resonance Theory Analysis

First, a theoretical analysis is carried out on the modal characteristics of the rectangular MPA to clarify how the seven selected modes can be properly regulated and jointly employed for wideband radiation. According to the cavity model, the resonant frequency of a rectangular microstrip patch antenna is written as [12]

(1) $$f_{mn}=\frac{c}{2\sqrt{\varepsilon_{eff}}}\sqrt{{\left(\frac{m}{L_{eff}}\right)}^{2}+{\left(\frac{n}{W_{eff}}\right)}^{2}}$$

where c is the speed of light in vacuum, *ε*_eff_ refers to the effective dielectric constant, while $L_{eff}$ and $W_{eff}$ indicate the effective patch length and width, respectively. Here, m and n are the modal orders along the x and y axes, respectively. Thus, different combinations of (m, n) correspond to different higher-order transverse magnetic modes [13].

Next, the mode selection is carried out. To realize dual-beam radiation, even-order modes are usually preferred because they can produce dual-beam patterns. Common modes that can produce dual-beam radiation patterns are summarized in Table 2. The symbol √ in the table indicates the seven finally selected modes. For bandwidth enhancement, the selected modes should be located close to each other in frequency, namely, with small frequency ratios relative to the reference mode. Since the average value between the lowest and highest resonant frequencies is about 6.73 GHz, TM_24_, whose resonant frequency is close to this value, is selected as the reference mode. As a result, we selected seven modes: TM_60_, TM_62_, TM_04_, TM_24_, TM_80_, TM_44_, and TM_82_, with their frequency ratios ranging from 0.8 to 1.2.

### 2.2. Bandwidth Enhancement Under Seven Modes

In this paper, we propose a broadband dual-beam antenna based on seven-mode resonance, and its main design guidelines are as follows.

(a) First, based on the frequency distribution of the common dual-beam modes, we select those with relatively close frequency ratios. In this paper, we have chosen the following seven modes, TM_04_, TM_24_, TM_44_, TM_62_, TM_82_, and TM_80_, because their frequency ratios lie between 0.8 and 1.2.

(b) Then, different slots are assigned according to the field distributions of different modes. The transverse slot is mainly used to tune TM_04_, TM_24_, and TM_44_ modes, while the longitudinal slots are mainly used to tune TM_62_, TM_82_, and TM_80_ modes.

(c) The outer longitudinal slots are introduced to improve the impedance matching among adjacent resonances, so that the redistributed modes can be merged into a continuous wide operating band.

**Figure 2:**
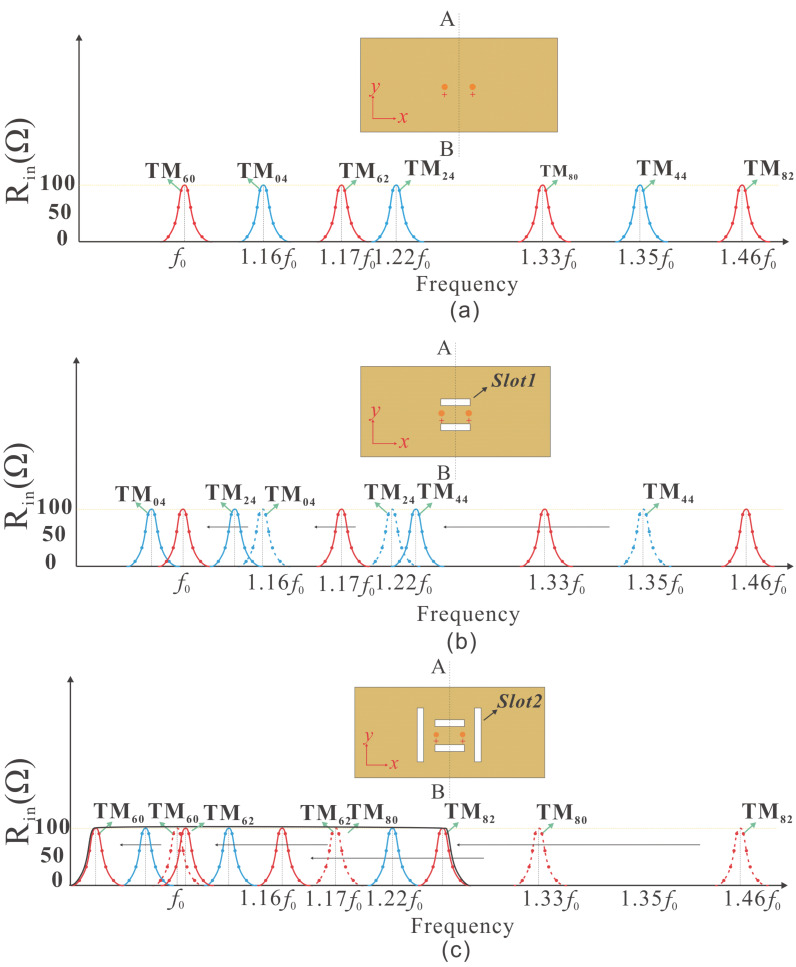
Evolution of resonant characteristics of the seven modes in three distinctive in-phase-fed MPAs. (**a**) Conventional structure. (**b**) Structure with Slot1. (**c**) Structure with Slot1 and Slot2.

To more clearly illustrate the evolution of the seven resonant modes in the proposed in-phase-fed MPA, Figure 2 schematically shows their resonant characteristics in three distinctive structures, namely, the conventional structure, the structure with Slot1, and the structure with Slot1 and Slot2. It can be observed that the resonant modes are gradually reallocated and brought close to each other as the slots are successively introduced, thereby creating the condition for wideband operation. Based on the above theoretical analysis, it can be found that if the six modes TM_04_, TM_24_, TM_44_, TM_62_, TM_82_, and TM_80_ can be gradually brought close to TM_60_ through proper structural perturbations, the seven modes can be continuously arranged over the desired operating frequency range and participate in radiation together, thereby significantly enhancing the impedance bandwidth [14]. Different from conventional single-mode or dual-mode patch antennas, the proposed structure does not rely on a single additional resonance [15], but on the combined distribution of multiple higher-order modes to form multiple resonant points. Therefore, it has a stronger potential for wideband operation.

In addition, owing to the in-phase feeding scheme adopted in this design, the field superposition of these modes in the main radiation direction is more consistent [16], which helps reduce the mutual cancellation among different modes and is favorable for maintaining stable radiation characteristics over a wide frequency band. Based on this idea, the proposed antenna is implemented through three successive steps to realize the frequency redistribution of the seven modes and the optimization of the input impedance.

### 2.3. Realization of Multi-Mode Bandwidth-Enhanced Antenna

On the basis of the preceding modal study, the resonant behavior of this in-phase-fed antenna is further examined to achieve effective bandwidth enhancement. Next, we will provide a detailed analysis of the various types of slots and conduct a parametric analysis aimed at evaluating the influence of key parameters on antenna performance.

**Figure 3:**
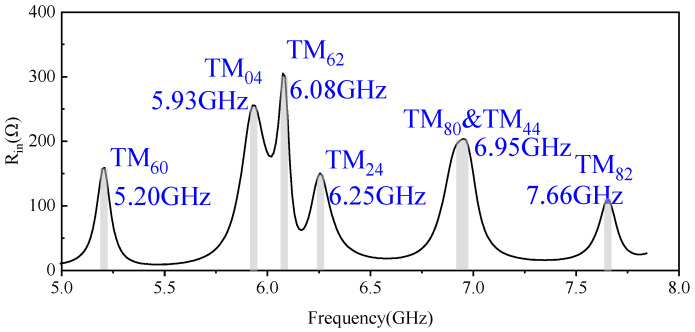
Z-parameters of the original rectangular patch without slots.

**Figure 4:**
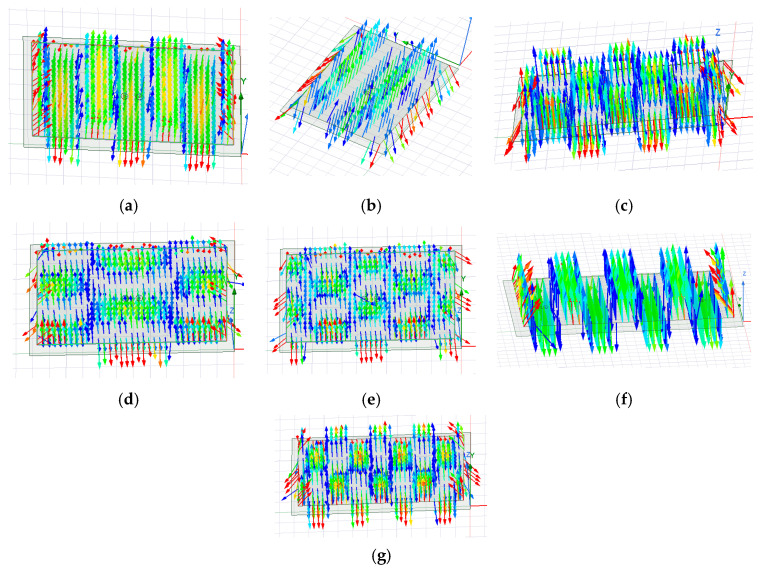
Electric-field distributions of the original rectangular patch without slots. (**a**) TM_60_ (5.2 GHz). (**b**) TM_04_ (5.9 GHz). (**c**) TM_62_ (6.0 GHz). (**d**) TM_24_ (6.2 GHz). (**e**) TM_44_ (6.9 GHz). (**f**) TM_80_ (6.9 GHz). (**g**) TM_82_ (7.6 GHz).

At the beginning, the active-Z response of the original rectangular patch without slots is first investigated, as shown in Figure 3. Seven resonant peaks can be observed in the considered frequency range, indicating that these higher-order modes can be excited in the original patch. Then, the corresponding electric-field distributions are shown in Figure 4 to further identify the modal orders. According to the resonant peaks and field distributions, these modes are identified as TM_60_, TM_04_, TM_62_, TM_24_, TM_44_, TM_82_, and TM_80_. The simulated field distributions are consistent with the modal features predicted by the rectangular-patch cavity model.

Based on the above modal verification, the design procedure of the proposed antenna is further clarified. First, TM_24_ is selected as the reference mode because its resonant frequency lies in the middle of the frequency range among the various dual-beam modes. Then, TM_04_, TM_60_, TM_62_, TM_44_, TM_82_, and TM_80_ are chosen according to the cavity-model frequency relation and their radiation-pattern compatibility. Second, different slots are assigned according to the field/current distributions of these modes. The transverse slot is mainly used to tune TM_04_, TM_24_, and TM_44_ modes, while the longitudinal slots are mainly used to tune TM_62_, TM_82_, and TM_80_ modes. Third, the outer longitudinal slots are introduced to improve the impedance matching among adjacent resonances, so that the redistributed modes can be merged into a continuous wide operating band.

*(1)*  *Reallocation of TM_04_, TM_24_, and TM_44_ Modes by Slot1*

**Figure 5:**
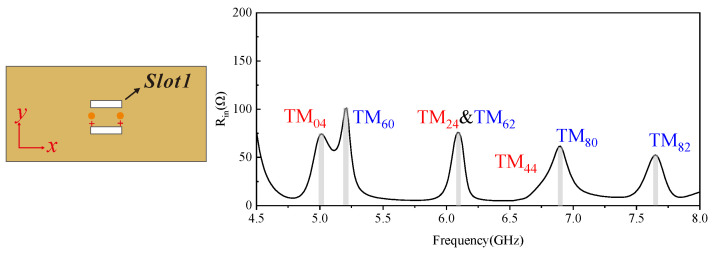
Geometry and active-Z response of the patch loaded with Slot1.

**Figure 6:**
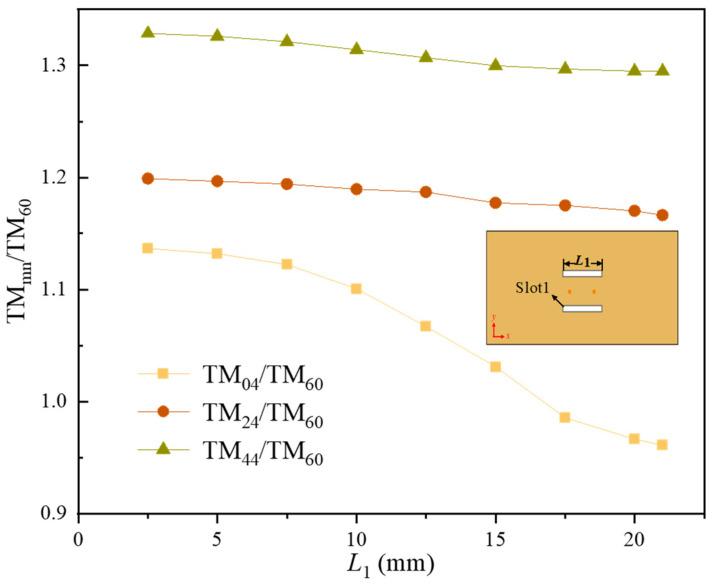
Frequency ratios (*f*_04_/*f*_60_), (*f*_24_/*f*_60_), and (*f*_44_/*f*_60_) as a function of *L*_1_ for the in-phase-fed MPA loaded with Slot1.

Firstly, a transverse slot, namely Slot1, is etched on the metallic patch. Figure 5 presents the active-Z response after introducing Slot1. And Figure 6 shows the variations in the frequency ratios of TM_04_, TM_24_, and TM_44_ with respect to TM_60_ versus the slot length. Here, the TM_60_ mode is selected as the reference because it resonates at the lowest frequency. It can be seen that all three curves decrease to different extents as the slot length increases, indicating that these three modes are gradually brought close to TM_60_ mode.

This suggests that Slot1 effectively extends the equivalent current paths associated with these modes, resulting in a continuous downward shift of the TM_04_, TM_24_, and TM_44_ resonant frequencies toward the TM_60_ mode. This also indicates that Slot1 mainly perturbs the strong-field regions of these three modes, while its effect on TM_60_ mode is relatively small. Therefore, an effective frequency reallocation of these three modes with respect to TM_60_ mode can be achieved.

More specifically, since Slot1 is transversely etched, its perturbation mainly acts on the modes with more complicated field distributions along the patch width. As a consequence, TM_04_, TM_24_, and TM_44_ are more sensitive to Slot1, whereas the resonant frequency variation in TM_60_ mode remains relatively limited. Hence, the ratios *f*_04_/*f*_60_, *f*_24_/*f*_60_, and *f*_44_/*f*_60_ keep decreasing.

*(2)*  *Reallocation of TM_62_, TM_82_, and TM_80_ Modes by Slot2*

**Figure 7:**
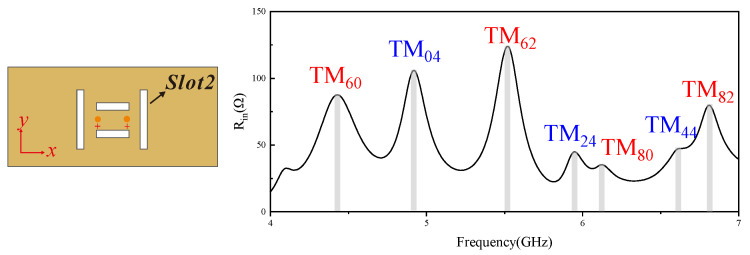
Geometry and active-Z response of the patch loaded with Slot1 and Slot2.

**Figure 8:**
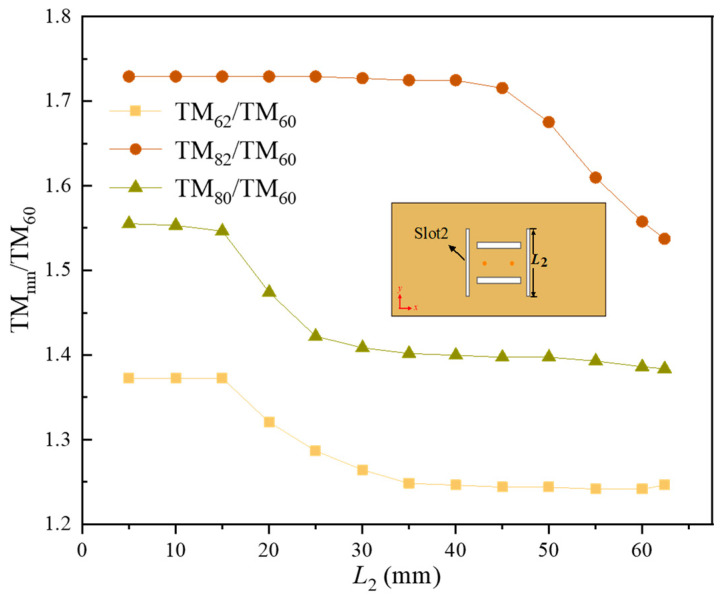
Frequency ratios (*f*_62_/*f*_60_), (*f*_82_/*f*_60_), and (*f*_80_/*f*_60_) as a function of *L*_2_ for the in-phase-fed MPA loaded with Slot2.

Subsequently, two longitudinal slots, denoted as Slot2, are additionally formed in the metallic patch. Figure 7 presents the active-Z response after introducing Slot1. And Figure 8 shows the variations in the frequency ratios of TM_62_, TM_82_, and TM_80_ modes with respect to TM_60_ mode versus the slot length. It can be observed that TM_82_ rapidly approaches TM_60_ mode when the slot length is larger than 40 mm, while TM_62_ and TM_80_ modes rapidly approach TM_60_ when the slot length is larger than 15 mm. However, when the slot length is further increased beyond 40 mm, the resonant frequencies of TM_62_ and TM_80_ modes remain almost unchanged.

From the physical point of view, since Slot2 is longitudinally etched, it mainly changes the equivalent electrical lengths of the modes related to the longitudinal current components. Therefore, it has a more significant effect on TM_62_, TM_82_, and TM_80_ modes. As the slot length increases, the current paths of these modes become significantly longer, leading to rapid decreases in their resonant frequencies. When the slot length increases to a certain value, however, the current distributions gradually become stable, and thus the frequency variations in TM_62_ and TM_80_ modes tend to saturate. Through this process, these modes originally located on the higher-frequency side are further brought close to TM_60_ mode, thereby creating the condition for the joint radiation of the seven modes.

*(3)*  *Impedance Matching Improvement by Slot3*

**Figure 9:**
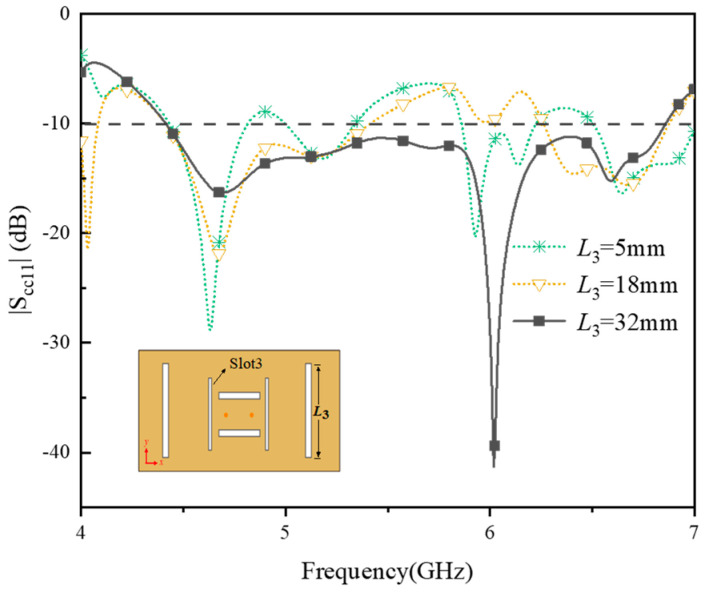
Simulated |S_cc11_| as a function of frequency under three different *L*_3_ values for the MPA loaded with Slot3.

Finally, an additional pair of longitudinal slots, denoted as Slot3, are loaded onto the metal patch to improve the antenna impedance matching. The influence of Slot3 length on the active S-parameter is illustrated in Figure 9. It is found that increasing the slot length leads to a noticeable improvement in the matching characteristic over several frequency intervals. Specifically, the active S-parameter falls below −10 dB within 4.8–5.0 GHz, 5.3–5.9 GHz, and 6.3–6.5 GHz. These results demonstrate that Slot3 plays an effective role in optimizing the impedance characteristic under multi-mode operation.

From the physical point of view, the loading of Slot3 further adjusts the equivalent reactance distribution around the feeding region and, hence, reduces the inductive component in the input impedance while improving the transition characteristics among adjacent resonant points. As the slot length gradually increases, the impedance matching among multiple modes becomes more coordinated. Therefore, the proposed antenna finally realizes a wideband performance from 4.4 to 6.9 GHz. It is thus clear that Slot3 is not mainly used to reallocate the positions of the resonant modes, but rather to optimize the impedance matching under the joint radiation of multiple modes, thereby providing a necessary condition for the realization of a wide impedance bandwidth.

## 3. Results

**Figure 10:**
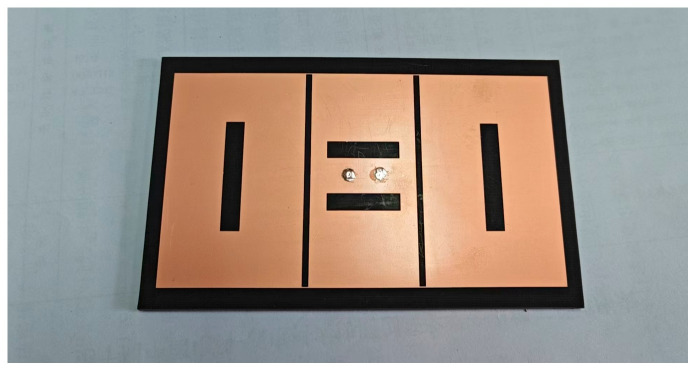
Photograph of the fabricated in-phase-fed MPA.

**Figure 11:**
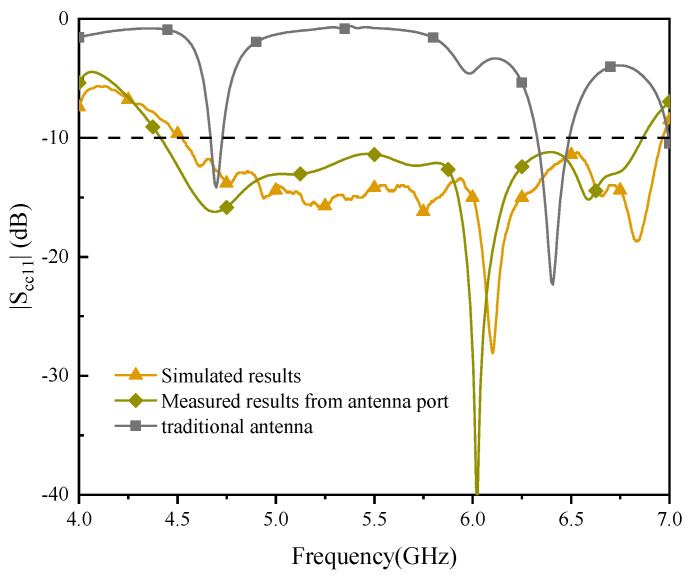
Simulated and measured |S_cc11_| of the proposed wideband antenna port, in comparison with the traditional antenna.

For verification of the operating mechanism and predicted performance of the proposed antenna, an antenna sample is manufactured and experimentally characterized. Figure 10 presents a photograph of the fabricated in-phase-fed MPA. During the experiment, the four-port scattering parameters, including S_11_, S_12_, S_21_, and S_22_, are acquired using a vector measurement instrument. Accordingly, the calculated and experimental |S_cc11_| responses for the antenna are determined from 0.5 × (S_11_ + S_12_ + S_21_ + S_22_), and the corresponding results are plotted in Figure 11, where the dashed line in Figure 11 represents the standard −10 dB for reference.

A close correspondence between the simulated and measured |S_cc11_| responses is observed across the operating band. Moreover, the measurement confirms that the antenna provides the expected impedance matching characteristic. The slight mismatch observed in the simulated and experimental results primarily arises from manufacturing tolerances and unavoidable uncertainties in the experimental setup.

**Figure 12:**
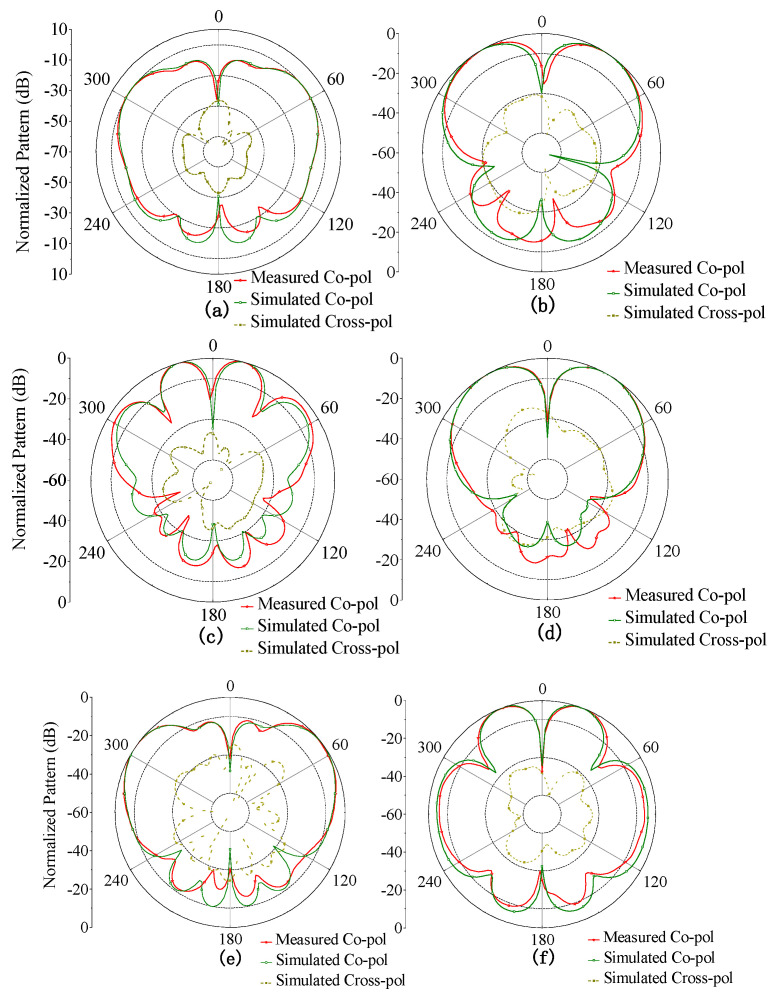
Simulated and measured radiation patterns of the proposed antenna. (**a**) XZ plane at 4.40 GHz. (**b**) YZ plane at 4.40 GHz. (**c**) XZ plane at 5.20 GHz. (**d**) YZ plane at 5.20 GHz. (**e**) XZ plane at 6.00 GHz. (**f**) YZ plane at 6.00 GHz.

Furthermore, the radiation patterns, gain response, and radiation efficiency of the antenna are experimentally investigated. As shown in Figure 12, the measured radiation patterns in the XZ and YZ planes at 4.4, 5.2, and 6.0 GHz agree well with the simulated results, and stable dual-beam radiation is maintained across the operating band. The antenna also achieves a cross-polarization discrimination of about 30 dB and a radiation efficiency higher than 95%.

**Figure 13:**
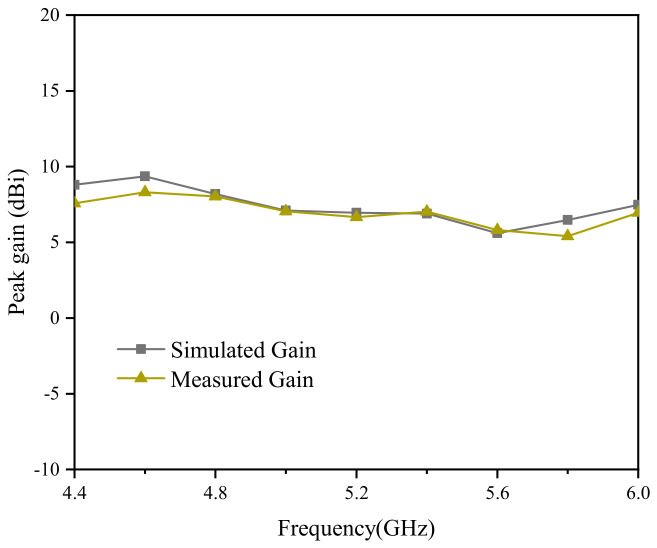
Simulated and measured peak gains versus frequency of the proposed antenna.

Figure 13 presents the calculated and tested gain responses of the antenna versus frequency. The tested data in Figure 13 are synthesized from the measured results of the two ports. The tested gain follows the calculated result closely across the operating band. The minor deviation is mainly attributed to fabrication tolerance and measurement loss. In addition, due to fabrication tolerance, the actual dielectric loss tangent of the substrate may be larger than that used in the simulation, which results in a relatively lower measured gain. Overall, good consistency between calculation and experiment is achieved for |S_cc11_|, radiation characteristics, and gain performance, confirming the validity of the proposed antenna configuration.

## 4. Discussion

The experimental results confirm the effectiveness of the antenna design methodology. The proposed antenna realizes bandwidth enhancement by properly redistributing seven higher-order resonant modes within a single patch resonator, rather than introducing additional radiators or complicated feeding structures. Therefore, a much broader operating bandwidth is achieved, while the antenna profile is still kept low.

Another important feature is that stable radiation performance is maintained over the whole operating band. Although multiple resonant modes are involved, the proposed in-phase-fed configuration can effectively control the radiation characteristics of these modes. As a result, stable dual-beam radiation patterns are obtained from 4.4 to 6.9 GHz, which indicates that the proposed design can not only broaden the impedance bandwidth but also preserve the desired radiation behavior.

It should also be noted that the three-slot structure plays different roles in the design. The transverse slot and longitudinal slots are mainly used to redistribute the resonant frequencies of the seven modes, while the outer longitudinal slots are mainly introduced to improve the impedance matching among adjacent resonances. In this way, the wideband performance is achieved through the combined effect of modal redistribution and impedance optimization.

Despite the minor deviations between simulation and measurement, these differences mainly arise from manufacturing tolerance and experimental uncertainty. Future studies may apply this design approach to additional frequency ranges and antenna array implementations for broader practical applications.

To further highlight the advantages of the proposed antenna, a comprehensive comparison with previously reported antennas is provided in Table 3. Most reported low-profile multi-mode patch antennas employ only two to four resonant modes and achieve bandwidths of 5.1–29.4%. In contrast, the proposed antenna utilizes seven resonant modes and achieves the widest bandwidth of 43.4% with a low profile of 0.056λ_0_. Although its peak gain of 9.9 dBi is slightly lower than that of [17], the proposed antenna provides a much wider bandwidth while maintaining a comparable gain level. Moreover, its radiation efficiency is higher than 95% across the operating band, which is higher than most compared antennas. The proposed antenna also exhibits competitive radiation-pattern quality, with sidelobe levels of about 16.6 dB in the XZ plane and 25 dB in the YZ plane. In addition, an average cross-polarization discrimination of about 30 dB is achieved, which is better than most reported values. Therefore, the proposed antenna demonstrates a balanced performance in terms of bandwidth, profile, gain, efficiency, sidelobe level, and cross-polarization.

## 5. Conclusions

This paper presents a compact broadband in-phase-fed MPA based on the cooperative operation of seven resonant modes, namely TM_60_, TM_04_, TM_62_, TM_24_, TM_44_, TM_82_, and TM_80_. First, the operating mechanism of the rectangular patch radiator was analyzed. Then, by loading one transverse slot and two pairs of longitudinal slots onto the patch, the seven resonant modes were progressively shifted toward each other in the frequency domain. Meanwhile, the impedance matching characteristic was further enhanced through proper optimization of the slot configuration. With this design, the antenna obtained a broad impedance bandwidth under the joint operation of the seven modes. Finally, an antenna prototype was manufactured and experimentally characterized, and the experimental results agreed well with the simulated predictions. The obtained results indicate that the proposed design operates from 4.4 to 6.9 GHz, providing an impedance bandwidth about 25 times wider than that of the conventional counterpart while maintaining stable dual-beam radiation performance. In addition, the antenna achieves a cross-polarization discrimination of about 30 dB and a radiation efficiency of higher than 95%, indicating good radiation performance. Therefore, the effectiveness of the proposed design is well confirmed.

## Figures and Tables

**Table 1:** Dimensions of in-fed antenna in Figure 1.

**Parameters**	***L***	***L*_1_**	***L*_2_**	***L*_3_**	***L*_g_**	***W***	***W*_1_**	***W*_2_**
Values(mm)	113	21	62.4	32	122	63	5	1.8
**Parameters**	***W*** **_3_**	***W*** **_g_**	***S*** **_1_**	***S*** **_2_**	***S*** **_3_**	***d***	***H***	***R***
Values(mm)	5.4	74.4	20.4	30.4	69.2	8.4	3	0.65

**Table 2:** Resonant frequency distribution of common dual-beam modes.

**Mode**	**TM_20_**	**TM_02_**	**TM_40_**	**TM_22_**	**TM_42_**
Frequency (GHz)	1.76	3.10	3.53	3.57	4.70
*f*_mn_/*f*_24_	0.27	0.47	0.54	0.55	0.72
**Mode**	**TM_60_**	**TM_62_**	**TM_04_**	**TM_24_**	**TM_80_**
Frequency (GHz)	5.30	6.15	6.21	6.46	7.07
*f*_mn_/*f*_24_	0.82(√)	0.95(√)	0.96(√)	1(√)	1.09(√)
**Mode**	**TM_44_**	**TM_82_**	**TM_64_**	**TM_06_**	**TM_84_**
Frequency (GHz)	7.15	7.72	8.17	9.32	9.41
*f*_mn_/*f*_24_	1.10(√)	1.19(√)	1.26	1.44	1.45
**Mode**	**TM_26_**	**TM_46_**	**TM_66_**	**TM_86_**	
Frequency (GHz)	9.49	9.97	10.73	11.70	
*f*_mn_/*f*_24_	1.46	1.54	1.66	1.81	

**Table 3:** Comparison with representative low-profile multi-mode patch antennas.

Ref.	Number of Modes	Bandwidth	Profile	Gain	Efficiency	SLL	Cross-Polarization
[17]	2	8.5%	0.03λ0	10.5 dBi	94%	21 dB	18 dB
[18]	4	11%	0.02λ0	9.21 dBi	91.6%	NG	NG
[19]	3	12.8%	0.03λ0	8.87 dBi	86%	5 dB	NG
[20]	3	29.4%	0.03λ0	6.2 dBi	80%	10 dB	20 dB
[21]	3	26.5%	0.05λ0	8 dBi	>0%	11 dB	23 dB
[22]	2	5.1%	0.02λ0	7.53 dBi	NG	10 dB	20 dB
This work	7	43.4%	0.056λ0	9.9 dBi	>95%	16.6 dB	30 dB

## Data Availability

The original contributions presented in this study are included in the article. Further inquiries can be directed to the corresponding author.

## Abbreviations

The following abbreviations are used in this manuscript:

MPA	microstrip patch antenna
